# Single-cell analyses revealed key tumor-infiltrating myeloid cell subsets associated with clinical outcomes in different subtypes of breast cancer

**DOI:** 10.1016/j.gendis.2023.101185

**Published:** 2023-11-30

**Authors:** Dingjie Wu, Si Chen, Yijin Liu, Bowen Yang, Ruixin Li, Feng Jin, Fan Yao, Yue Fang

**Affiliations:** a Department of Microbial and Biochemical Pharmacy, School of Pharmacy, China Medical University, Shenyang, Liaoning 110122, China; b Department of Breast Surgery and Surgical Oncology, Research Unit of General Surgery, The First Affiliated Hospital of China Medical University, Shenyang, Liaoning 110000, China; c Department of Medical Oncology, The First Affiliated Hospital of China Medical University, Shenyang, Liaoning 110000, China

Breast cancer is the most common cancer and the leading cause of cancer death in women worldwide.^1^ Tumor-infiltrating myeloid cells (TIMs), key components of tumor microenvironment, are considered to be potential therapeutic targets for cancer recently,^2^^,^^3^ however, their heterogeneity remains insufficiently characterized in different breast cancer subtypes. A more detailed TIM transcriptional atlas across breast cancer subtypes at the single-cell level is required to better understand the underlying mechanisms influencing the prognosis of breast cancer patients. Here, we painted the landscapes of TIMs and investigated the diversity of TIM subsets across breast cancer subtypes. Particularly, we identified a novel subset, *CXCL10*^+^ conventional dendritic cells (cDCs), which are abundant in triple-negative breast cancer (TNBC) and have the ability to recruit tumor-associated macrophages (TAMs). We further discovered the developmental origins of *CXCL10*^+^ cDCs. Moreover, we identified novel subsets of macrophages and mast cells and systematically analyzed their characteristics associated with clinical outcomes in different breast cancer subtypes. Our findings provide valuable information for the identification of potential targets and biomarkers to better direct breast cancer immunotherapies.

To elucidate the intercellular heterogeneity of TIMs in breast cancer, we performed unsupervised graph-based clustering and identified five common major lineages, namely cDCs, plasmacytoid dendritic cells (pDCs), monocytes, macrophages, and mast cells based on canonical cell markers (Fig. 1A), which were further verified by specific signature gene expressions (Fig. S1). cDCs, monocytes, and macrophages were further divided into multiple sub-populations (Fig. 1B). Since the proportion of cDCs exhibited relatively fluctuation across breast cancer subtypes (Fig. S2A), we further characterized the heterogeneity and function of cDCs. Importantly, except for the clusters of *CLEC9A*^+^ cDC1s, *CD1C*^+^ cDC2s, and *LAMP3*^+^ cDCs,^2^ a discrete cluster of *CXCL10*^+^ cDCs, which was previously not reported in breast cancer, was identified based on top marker gene expression (Fig. 1C, D). Notably, expression of macrophage inflammatory proteins, including CCL2, CCL3, CCL4, and CCL5,^4^ were all up-regulated in tumor-derived *CXCL10*^+^ cDCs compared with non-tumor-derived cDCs (Fig. 1E), suggesting that tumor-derived *CXCL10*^+^ cDCs may have a capability of TAM recruitment in breast cancer tissues. Intriguingly, the proportion of *CXCL10*^+^ cDCs in TNBC was much higher than that in estrogen receptor-positive (ER+) tumors (Fig. S2B), and the number of macrophages in TNBC was highest among the three breast cancer subtypes (Fig. S2C, D). These results suggested that higher *CXCL10*^+^ cDCs in TNBC might elicit TAM accumulation which in turn promotes tumor growth. This might be one of the reasons for poorer prognosis in TNBC patients compared with patients with other breast cancer subtypes. Additionally, we found that PD-L1 and its regulators were universally up-regulated in tumor-derived *CXCL10*^*+*^ cDCs compared with non-tumor-derived cDCs (Fig. S2E), suggesting that *CXCL10*^+^ cDCs might cause T cells to differentiate into regulatory T cells via PD-1/PD-L1, thereby suppressing anti-tumor immunity. Collectively, *CXCL10*^+^ cDCs might be a potential therapeutic target in TNBC. Also, *CXCL10*^+^ cDCs might be isolated as a potential pool for loading tumor-associated antigens for cell-based therapy, which warrants further investigation.

**Figure 1:**
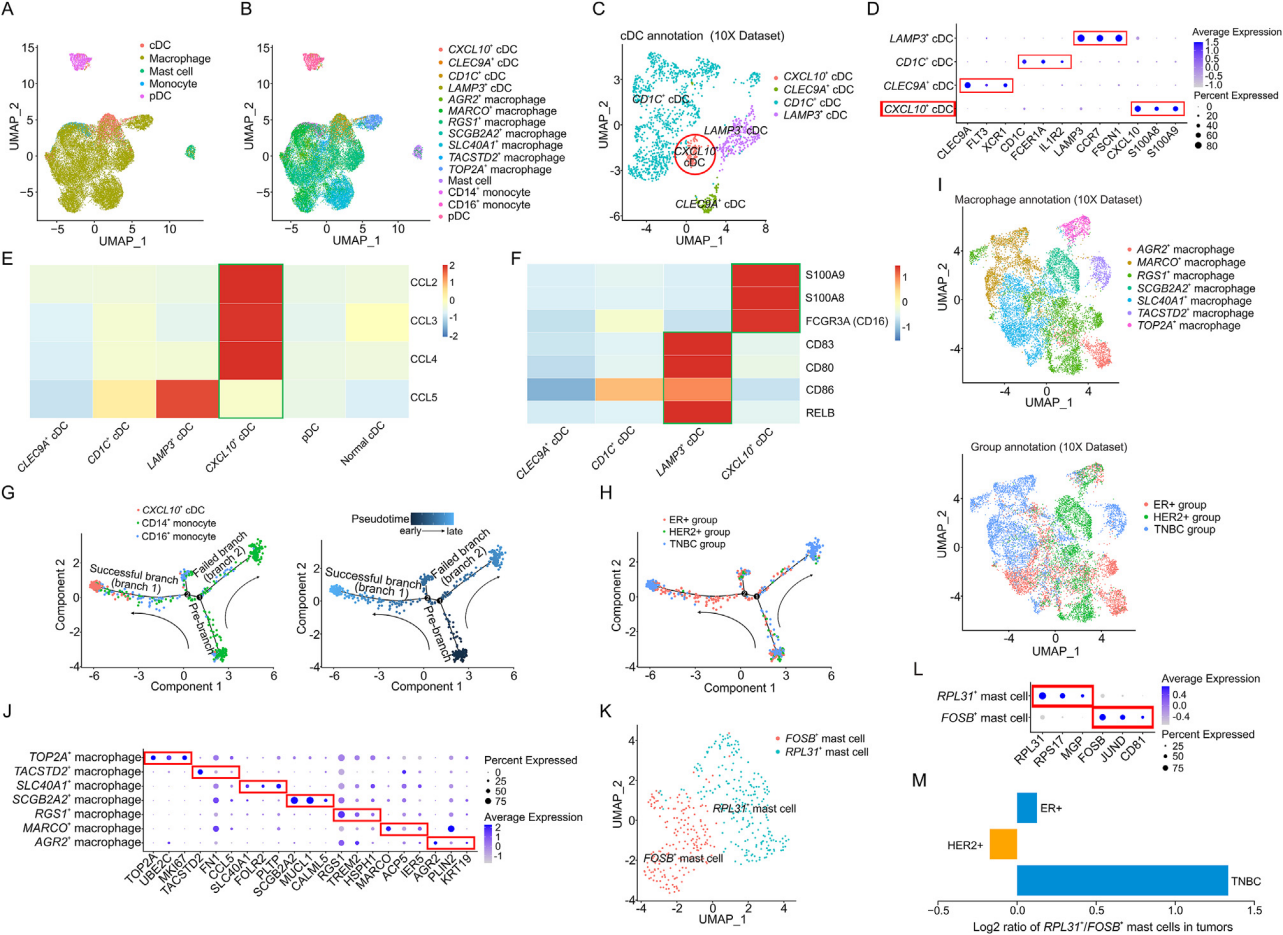
Identification of key tumor-infiltrating myeloid cell subsets in different breast cancer subtypes. **(A)** The UMAP plot showing the major lineages of myeloid cells originating from two independent datasets (10X and CITE Dataset) in breast cancer by Harmony algorithm. **(B)** The UMAP plot showing the subsets of myeloid cells originating from two independent datasets (10X and CITE Dataset) in breast cancer by Harmony algorithm. **(C)** The UMAP plot showing the major lineages of cDCs in breast cancer in 10X Dataset. **(D)** The bubble heatmap showing expression levels of selected cDC signature genes in breast cancer in 10X Dataset. Dot size indicates fraction of expressing cells, colored based on normalized expression levels. **(E)** The heatmap showing the tumor macrophage chemotactic factor markers for distinct dendritic cell subsets, colored based on normalized expression levels in 10X Dataset. **(F)** The heatmap showing the mature markers in distinct cDC subsets in 10X Datasets. **(G)** Developmental trajectory of *CXCL10*^+^ cDCs and monocytes (left) along with pseudotime (right) inferred by Monocle2 in 10X Dataset. **(H)** Developmental trajectory of *CXCL10*^+^ cDCs and monocytes annotated with three breast cancer subtypes in 10X Dataset. **(I)** The UMAP plot showing the major lineages of macrophages in breast cancer (top) and macrophages originating from three breast cancer subtypes (bottom) in 10X Dataset. **(J)** The bubble heatmap showing expression levels of selected macrophage signature genes in breast cancer in 10X Dataset. Dot size indicates fraction of expressing cells, colored based on normalized expression levels. **(K)** The UMAP plot showing the two major lineages of mast cells in breast cancer in 10X Dataset. **(L)** The bubble heatmap showing expression levels of selected mast cell signature genes in breast cancer in 10X Dataset. Dot size indicates fraction of expressing cells, colored based on normalized expression levels. **(M)** The bar plot showing the ratios of *RPL31*^+^ mast cells to *FOSB*^+^ mast cells in three breast cancer subtypes in 10X Dataset. cDCs, conventional dendritic cells.

Next, we investigated the developmental origins of *CXCL10*^+^ cDCs in breast cancer. In tumor-derived *CXCL10*^+^ cDCs, CD14^+^ monocyte markers (*S100A8*, *S100A9*) and CD16^+^ monocyte markers (*CD16*) were universally up-regulated compared with *CLEC9A*^+^, *CD1C*^+^, and *LAMP3*^+^ cDCs (Fig. 1F). Using Monocle2, we analyzed pseudotime trajectory between monocytes and *CXCL10*^+^ cDCs. Interestingly, the pseudotime trajectory began with monocytes in pre-branch and then split into *CXCL10*^+^ cDCs in branch 1 or monocytes in branch 2, indicating a successful transition from monocytes to *CXCL10*^+^ cDCs (Fig. 1G). We also performed gene expression dynamics profiling along the trajectories as well as gene ontology (GO) analysis, and identified markers or pathways differentially expressed or activated between monocytes and *CXCL10*^+^ cDCs (Fig. S3A, B). Additionally, developmental trajectories of *CXCL10*^+^ cDCs and monocytes in different breast cancer subtypes were analyzed (Fig. 1H; Fig. S3C). Interestingly, CD14^+^ monocytes were evenly distributed in the whole reprogramming, while CD16^+^ monocytes were concentrated in the middle of reprogramming (Fig. S3C). Intriguingly, the number of CD16^+^ monocytes and the average proportion of CD16^+^ monocytes among all monocytes in TNBC were significantly lower than those in ER+ or human epidermal growth factor receptor 2-positive (HER2+) tumors (Fig. S3D, E). These results suggested that lower CD16^+^ monocytes in TNBC might be linked with a poorer prognosis of TNBC than other breast cancer subtypes. The differential distributions of CD14^+^ versus CD16^+^ monocytes and the variation in the number of CD16^+^ monocytes between different subtypes of breast cancer might be image-based data valued for machine learning for future diagnosis index, which needs more investigation.

We next sought to explore the heterogeneity of macrophages in breast cancer. Seven and six macrophage subsets were identified from 10X and CITE Dataset, respectively (Fig. 1I; Fig. S4A). The top three marker genes in each subset as well as the proportion of each subset were shown in Figure 1J and Figure S4B and C. Considering the correlation between *CD163* (M2 TAM marker) and *MARCO* expression (Fig. S4D) as well as survival analysis results (Fig. S4E), we speculate that *MARCO*^+^macrophages might be an indicator of worse prognosis in ER + or HER2+ subtype, but play an unidentified role in TNBC. Additionally, gene expression analysis and survival analysis (Fig. S4F, G) suggested that *SLC40A1*^+^ macrophages might serve as an indicator of better prognosis in ER+ and TNBC patients. The associations of *RGS1* expression with the proportion of M2 TAMs, plasma cells, and naive B cells, as well as overall survival (Fig. S5A, B) suggested that *RGS1*^+^ macrophages might be an indicator of worse prognosis in the ER+ subtype. *AGR2*^+^, *SCGB2A2*^+^, and *TACSTD2*^+^ macrophages were mainly originated from ER+, HER2+, and TNBC, respectively, whereas *TOP2A*^+^ macrophages were originated from all subtypes (Fig. S4C). Interestingly, higher expression of these macrophage markers was associated with worse outcomes of ER+, HER2+, TNBC, and all subtypes, respectively (Fig. S5C, D), suggesting that these four subsets might be indicators of worse prognosis in their respective breast cancer patients.

We also analyzed the proportion of myeloid cells in breast cancer using bulk RNA-seq datasets and found that it was similar to that of scRNA-seq datasets except for mast cells showing fluctuation in bulk RNA-seq datasets (Fig. S2A; Fig. S6A). Then, we further investigated the heterogeneity of mast cells in breast cancer. The mast cells in breast cancer were divided into *FOSB*^+^ and *RPL31*^+^ subsets (Fig. 1K, L). Strikingly, ER+ and TNBC subtypes exhibited significantly higher *RPL31*^+^ subset than *FOSB*^+^ subset, whereas the HER2+ subtype exhibited a higher *FOSB*^+^ subset than *RPL31*^+^ subset (Fig. 1M), indicating that the imbalance of these two subsets might influence the prognosis of breast cancer patients. To explore it, we analyzed the differentially expressed genes of *RPL31*^+^ subset compared with *FOSB*^+^ subset in breast cancer as well as mast cells in normal breast tissues, respectively (Fig. S6B, C). GO analysis showed that the down-regulated genes in *RPL31*^+^ subset shown in Figure S6B and C were majorly involved in “negative regulation of apoptotic signaling pathway” (Fig. S6D, E), suggesting that *RPL31*^+^ subset might promote apoptosis of tumor cells and activate the immune response to limit pro-tumor effects of immune cells in breast cancer, whereas *FOSB*^+^ subset might have an opposite effect. This might be the reason that the imbalance of these two mast cell subsets affects prognosis. Survival analysis showed that higher expression of *TPSAB1*, a gene marker for mast cells,^5^ was associated with a survival advantage in ER+ patients, whereas an opposite effect was observed in HER2+ patients (Fig. S6F). Although higher *TPSAB1* expression associated with survival advantage was observed in TNBC patients (Fig. S6F), extremely lower mast cell proportion in TNBC exhibited lower *TPSAB1* expression, which might be linked with poorer prognosis of TNBC patients compared with ER+ subtype. Collectively, our results suggested that *RPL31*^+^ mast cells might be an indicator of better prognosis in ER+ breast cancer patients.

In conclusion, our study illuminated key TIM subsets associated with clinical outcomes in different breast cancer subtypes. Our data presented here may help identify better molecular targets for prognosis and treatment and provide new insights into the immunotherapeutic strategies most appropriate for each subtype of breast cancer.

## Funding

This work was supported by the National Natural Science Foundation of China (No. 81974418), Basic Research Project of Liaoning Province (No. JC2019002), Doctoral Start -up Foundation of Liaoning Province (No. 2019-BS-284), and Youth backbone Support Program of China Medical University (No. QGZ2018081).

## Conflict of interests

All authors declare that the research was conducted in the absence of any commercial or financial relationships that could be construed as a potential conflict of interest.

## Figures and Tables

**Figure S1:**
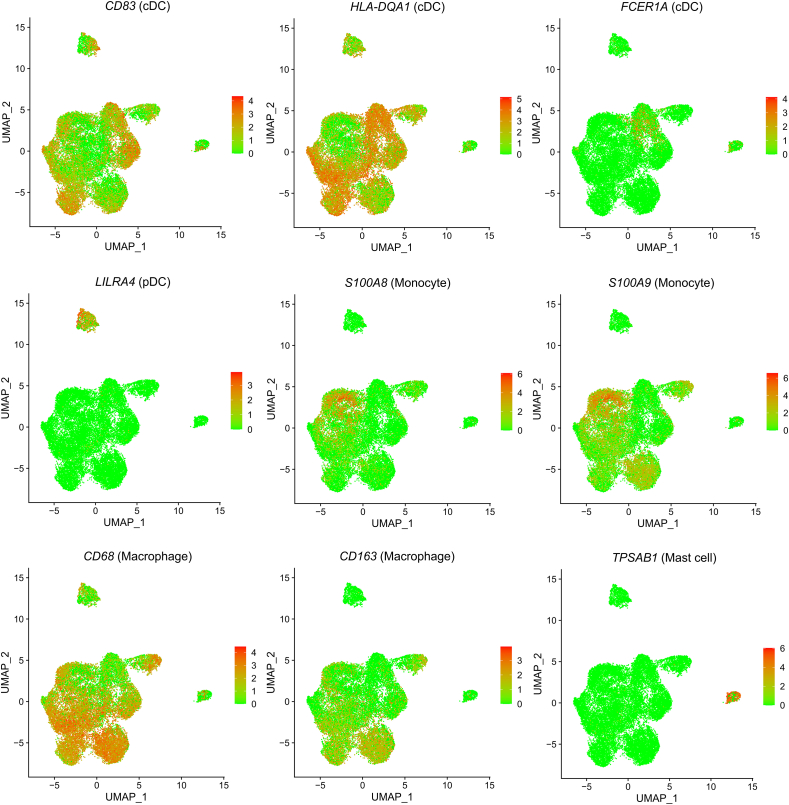
Identification of myeloid cell subset markers in breast cancer. The UMAP plots show the marker gene expression for the major lineages of myeloid cells originating from two independent datasets (10X and CITE Dataset) in breast cancer by Harmony algorithm.

**Figure S2:**
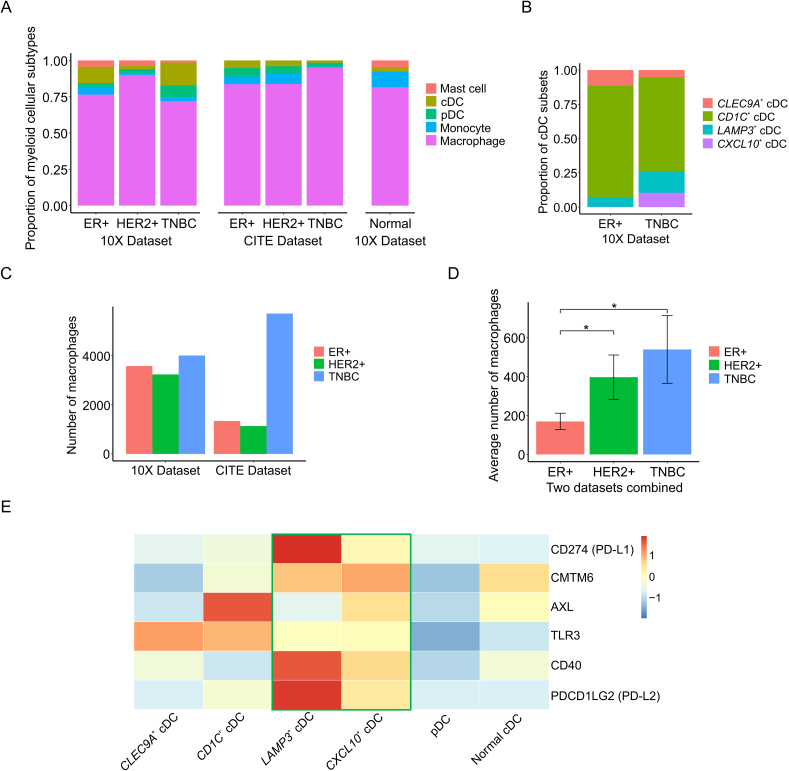
Characteristics of myeloid cell subsets in different breast cancer subtypes. **(A)** The fraction of major lineages of myeloid cells originating from three subtypes of breast cancer tissues across two datasets and normal breast tissues in 10X Dataset. **(B)** The fraction of major lineages of cDCs originating from estrogen receptor-positive (ER+) breast cancer and TNBC in 10X Dataset. **(C)** The bar plot showing the number of macrophages collected from three breast cancer subtypes across two datasets. **(D)** The bar plot showing the average number of macrophages in three breast cancer subtypes across two combined tumor tissue datasets (10X and CITE Dataset). Error bars indicate the mean standard error for the calculated mean value; *P < 0.05, using two-sided Wilcoxon test. **(E)** The heatmap showing the PD-L1 related markers for distinct dendric cell subsets, colored based on normalized expression levels in 10X Dataset.

**Figure S3:**
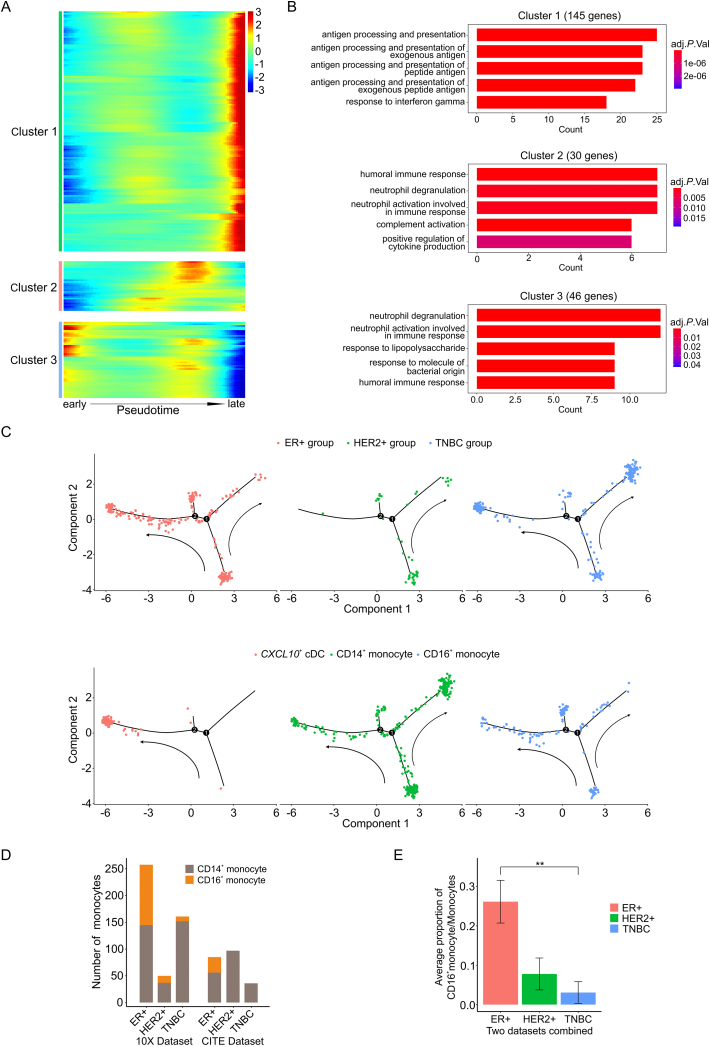
Gene expression dynamics profiling along pseudotime trajectories in different breast cancer subtypes. **(A)** Gene expression heatmap of differentially expressed genes cataloged in three clusters in a pseudo-temporal order. **(B)** Gene ontology analyses of each gene cluster (cluster1, 2, and 3). **(C)** Developmental trajectory annotated with each subtype of breast cancer (top), or annotated with *CXCL10*^+^ cDCs, CD14^+^ monocytes, and CD16^+^ monocytes, respectively (bottom) in 10X Dataset. **(D)** The number of monocyte subsets originating from three breast cancer subtypes in 10X and CITE Dataset. **(E)** The bar plot showing the average proportion of CD16^+^ monocytes/monocytes in three breast cancer subtypes across two combined tumor tissue datasets (10X and CITE Dataset). Error bars indicate the mean standard error for the calculated mean value; **P < 0.01, using two-sided Wilcoxon test.

**Figure S4:**
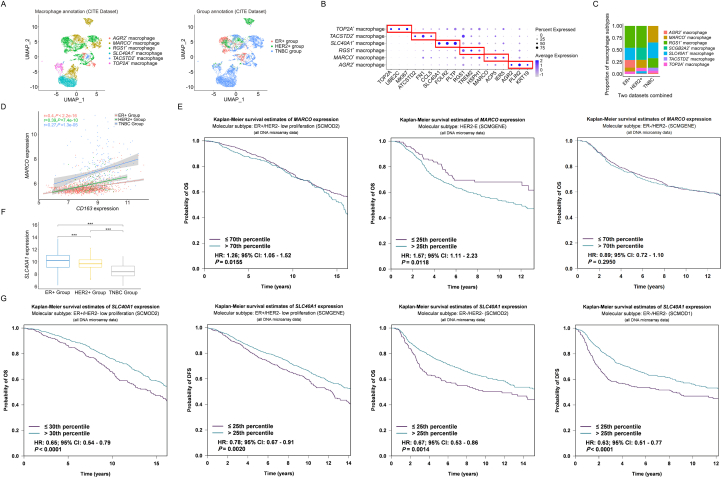
Characteristics of macrophage subsets in three breast cancer subtypes (part one). **(A)** The UMAP plot showing the major lineages of macrophages in breast cancer (left) and macrophages originating from three breast cancer subtypes (right) in the CITE Dataset. **(B)** The bubble heatmap showing expression levels of selected macrophage signature genes in breast cancer in CITE Dataset. Dot size indicates the fraction of expressing cells, colored based on normalized expression levels. **(C)** The fraction of major lineages of macrophages originating from three breast cancer subtypes across two combined tumor tissue datasets (10X and CITE Dataset). **(D)** Correlation of *MARCO* expression with the *CD163* expression across three breast cancer subtypes in the METABRIC dataset. **(E)** Breast Cancer Gene-Expression Miner v4.7 shows worse clinical outcomes in estrogen receptor-positive (ER+) (the first) and human epidermal growth factor receptor 2-positive (HER2+) breast cancer patients (the second) with the higher expression of *MARCO* in OS, and no association of OS with *MARCO* expression in TNBC patients (the third). Cox regression. **(F)** Expression of *SLC40A1* in three breast cancer subtypes in the METABRIC dataset. **(G)** Breast Cancer Gene-Expression Miner v4.7 shows better clinical outcomes in ER + breast cancer patients with the higher expression of *SLC40A1* in OS (the first) and DFS (the second), and in TNBC patients with the higher expression of *SLC40A1* in OS (the third) and DFS (the fourth). Cox regression. HR, hazard ratio; OS, overall survival; DFS, disease-free survival.

**Figure S5:**
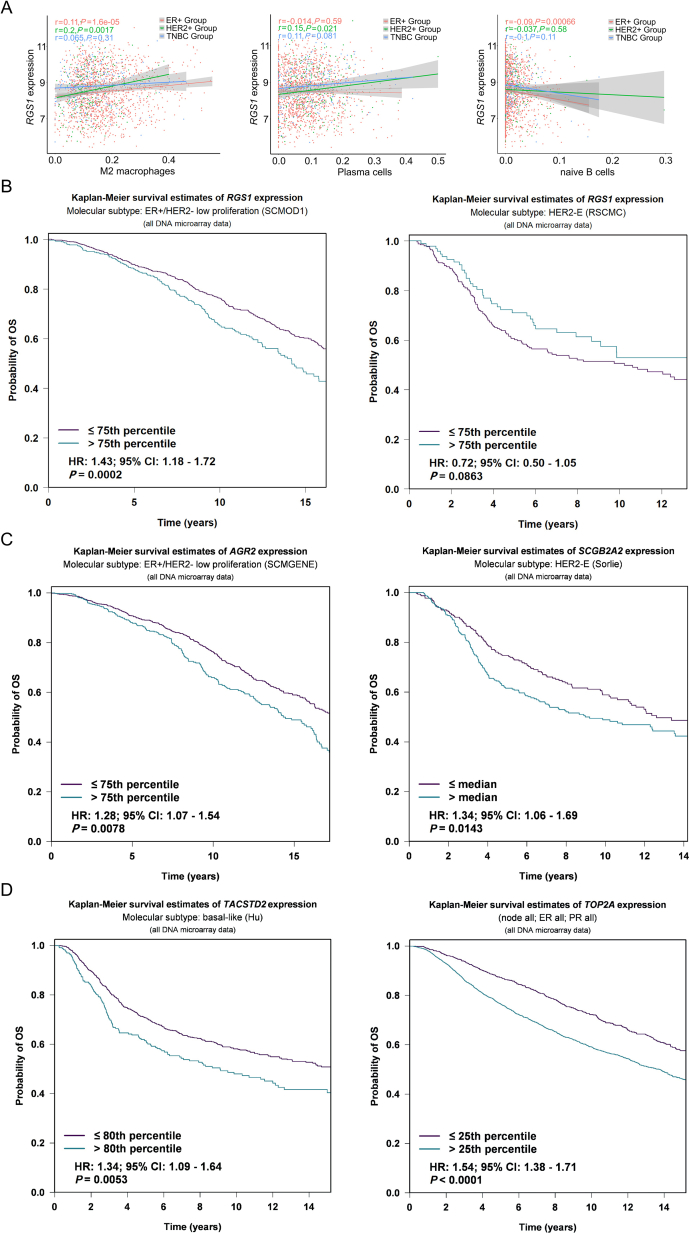
Characteristics of macrophage subsets in three breast cancer subtypes (part two). **(A)** Correlation of *RGS1* expression with the M2 macrophages (the first), the plasma cells (the second), and the naïve B cells (the third) across three breast cancer subtypes in the METABRIC dataset. **(B)** Breast Cancer Gene-Expression Miner v4.7 shows worse clinical outcomes in estrogen receptor-positive (ER+) breast cancer patients with the higher expression of *RGS1* in OS (left) and no association of OS with *RGS1* expression in human epidermal growth factor receptor 2-positive (HER2+) breast cancer patients (right). Cox regression. **(C)** Breast Cancer Gene-Expression Miner v4.7 shows worse clinical outcomes in ER + breast cancer patients with a higher expression of *AGR2* (left) and in HER2+ breast cancer patients with a higher expression of *SCGB2A2* (right). Cox regression. **(D)** Breast Cancer Gene-Expression Miner v4.7 shows worse clinical outcomes in TNBC patients with a higher expression of *TACSTD2* (left) and in total breast cancer patients with a higher expression of *TOP2A* (right). Cox regression. HR, hazard ratio; OS, overall survival.

**Figure S6:**
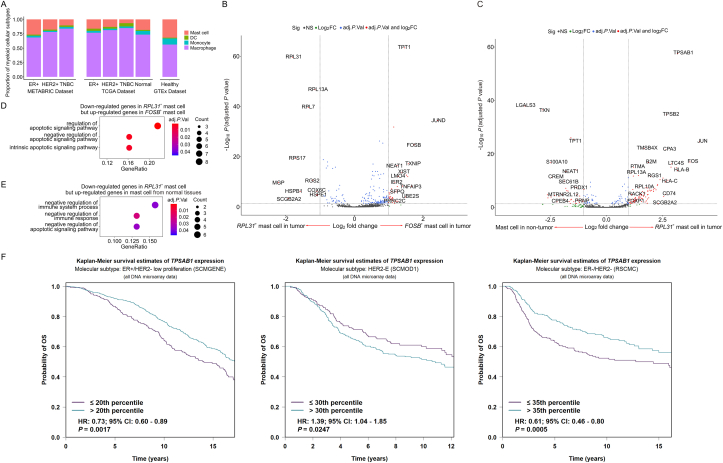
The imbalance of mast cell subsets in three breast cancer subtypes. **(A)** The fraction of four major lineages of myeloid cells estimated by CIBERSORT in three subtypes of breast cancer tissues from the METABRIC and TCGA dataset, normal breast tissues from the TCGA dataset, and healthy breast tissues from the GTEx dataset. **(B)** The volcano plot showing differentially expressed genes (DEGs) between *FOSB*^+^ and *RPL31*^+^ mast cells in breast tumors in 10X Dataset. Adjusted *P* value < 0.05. **(C)** The volcano plot showing DEGs between *RPL31*^+^ from breast tumors and mast cells from normal tissues in breast cancer patients in 10X Dataset. Adjusted *P* value < 0.05. **(D)** Gene ontology analyses of down-regulated gene cluster in *RPL31*^+^ mast cells but up-regulated in *FOSB*^+^ mast cells in breast tumors in 10X Dataset. **(E)** Gene ontology analyses of down-regulated gene cluster in *RPL31*^+^ mast cells from breast tumors but up-regulated in mast cells from normal tissues in 10X Dataset. **(F)** Breast Cancer Gene-Expression Miner v4.7 shows better clinical outcomes in estrogen receptor-positive (ER+) breast cancer patients (the first) or TNBC patients (the third) with the higher expression of mast cell marker (*TPSAB1*), but poor clinical outcomes in human epidermal growth factor receptor 2-positive (HER2+) breast cancer patients with the higher expression of mast cell marker (*TPSAB1*) (the second). Cox regression. HR, hazard ratio.
