# Supplementary material for: Single-cell analyses revealed key tumor-infiltrating myeloid cell subsets associated with clinical outcomes in different subtypes of breast cancer
Source: Genes Dis. 2023 Nov 30;11(6):101185. doi: 10.1016/j.gendis.2023.101185 (PMC11298853; doi:10.1016/j.gendis.2023.101185)
Supplement: Multimedia component 1 [file mmc1.docx]

**Single-cell analyses revealed key tumor-infiltrating myeloid cell subsets associated with clinical outcomes in different subtypes of breast cancer**

**Materials and Methods**

**Single-cell RNA sequencing datasets processing**

10X and CITE Dataset of breast cancer in mRNA level can be obtained from Gene Expression Omnibus (GEO) database ^[^[^1^](#_ENREF_1)^]^ with the series accession number GSE161529 ^[^[^2^](#_ENREF_2)^]^ and GSE176078 ^[^[^3^](#_ENREF_3)^]^, respectively. For each dataset, we first distinguished between normal tissues and tumor tissues, stratifying tumor tissues into ER+ breast cancer, HER2+ breast cancer and triple-negative breast cancer (TNBC) ^[^[^4^](#_ENREF_4)^]^. Then, we obtained 13 adjacent non-tumor tissues as well as 58 tumor tissues from 58 patients diagnosed with three breast cancer subtypes, including ER+ (29 samples), HER2+ (11 samples), and TNBC (18 samples). The data of tumor tissue samples were obtained from 10X Dataset and CITE Dataset, while the data of adjacent non-tumor tissue samples were obtained from 10X Dataset only. After filtering expression matrices of low UMI and high mitochondrial gene cells and normalizing the data, we selected 2,000 highly-variable genes for downstream analysis by the R package Seurat ^[^[^5^](#_ENREF_5)^]^ (version 4.0.2). Preprocessed with ScaleData function, principal component analysis (PCA) was used to reveal the main axes of variation. For visualization, the dimensionality of three breast cancer subtypes across two datasets was further reduced using t-distributed Stochastic Neighbor Embedding (t-SNE) or Uniform Manifold Approximation and Projection (UMAP). For TNBC tumor tissues in 10X Dataset, we first performed unsupervised clustering and characterized immune cell clusters with high expression of *PTPRC* (*CD45*) by using FindAllMarkers function, and a second-round of dimension reduction and unsupervised clustering were performed to characterize myeloid cells following the above-described strategy. Myeloid cells were also characterized in other breast cancer subtypes in 10X and CITE Dataset following the above-described strategy. Thus, we collected a total of 22,776 myeloid cells derived from the tumors across two datasets, and 1,024 myeloid cells derived from adjacent non-tumor tissues in 10X Dataset. Lastly, we further divided cDCs, monocytes, and macrophages into multiple sub-populations by integrating the same major linage of myeloid cells from three breast cancer subtypes in 10X and CITE Dataset.

**Integration of 10X Dataset and CITE Dataset by Harmony**

To remove the batch effects within three breast cancer subtypes across two datasets, the R package Harmony ^[^[^6^](#_ENREF_6)^]^ (version 0.1.0), an algorithm that could identify and merge shared broad populations as well as fine-grained subpopulations among multiple datasets, was used to integrate the data from 10X Dataset and CITE Dataset. The results of Harmony integration from two datasets were sensitive and accurate. We further verified these five common major lineages, including cDCs, pDCs, monocytes, macrophages, and mast cells, by high expression of the specific signature genes, including *CD83*/*HLA*/*FCER1A*, *LILRA4*, *S100A8*/*S100A9*, *CD68*/*CD163*, and *TPSAB1*, respectively.

**Differential expression analysis**

In order to identify the differentially expressed genes (DEGs) between two specified groups of clusters, we used the R package EnhancedVolcano (version 1.11.3) to evaluate the fold change and *P*-value of each gene. Genes with adjusted *P*-value less than 0.05 and |log2FC| more than 1 were considered as DEGs.

**Developmental trajectory inference**

To characterize the development of *CXCL10*^+^ cDCs and monocytes, we first integrated the data of monocytes, including CD14^+^ monocytes and CD16^+^ monocytes, as well as *CXCL10*^+^ cDCs in 10X Dataset. Then, the R package Monocle ^[^[^7^](#_ENREF_7)^]^ (version 2.18.0) was used, and cell differentiation was inferred with default parameters as recommended by the Monocle developers after dimension reduction and cell ordering.

**Gene Ontology analysis**

Gene ontology (GO) term enrichment analyses were performed using the R package clusterProfiler ^[^[^8^](#_ENREF_8)^]^ (version 3.18.1). Terms with adjusted *P*-value less than 0.05 were defined as significantly enriched.

**Inference of infiltrating immune cells in bulk RNA-seq datasets**

To investigate the correlation of different TIM subset gene markers with TIM cell proportion across three breast cancer subtypes in the METABRIC dataset, Cell-type Identification By Estimating Relative Subsets Of RNA Transcripts (CIBERSORT) ^[^[^9^](#_ENREF_9)^]^ was performed. Using CIBERSORT, we also verified the proportion of major lineages of myeloid cells in bulk RNA-seq datasets. METABRIC and GTEx dataset were obtained from the public website ([cBioPortal for Cancer Genomics::Datasets](http://www.cbioportal.org/datasets)) and ([Genotype-Tissue Expression (GTEx) (nih.gov)](https://commonfund.nih.gov/GTEx/)), respectively. The fraction of immune cells data from The Cancer Genome Atlas (TCGA) breast cancer (BRCA) datasets were obtained from the public website (<https://gdc.cancer.gov/about-data/publications/panimmune>). The clinical data of TCGA were downloaded by using SangerBox (version 1.0.9). We used these data to evaluate the relative proportion of major lineages of myeloid cells among three breast cancer subtypes, normal and healthy breast tissues based on R (version 4.0.5).

**Breast Cancer Gene-Expression Miner v4.7 analysi***s*

Using Breast Cancer Gene-Expression Miner v4.7 (bc-GenExMiner v4.7) online dataset ([breast cancer gene expression database | bc-GenExMiner (unicancer.fr)](http://bcgenex.ico.unicancer.fr/BC-GEM/GEM-Accueil.php?js=1)), the association between TIM subset gene markers and the prognosis of breast cancer patients was evaluated. In survival analysis, samples were divided into high and low groups based on optimal percentile of gene expression by using all DNA microarrays dataset in bc-GenExMiner v4.7. *P* < 0.05 was considered statistically significant.

**Statistical analysis**

The survival analysis was based on web-based application called bc-GenExMiner v4.7. Other statistical analyses were performed in R (version 4.0.5). The gene expression in three breast cancer subtypes was calculated using two-sided Wilcoxon test. Gene correlation analysis in the METABRIC dataset was calculated using Pearson correlation. *P* < 0.05 was considered statistically significant.

**REFERENCES**

1. Davis S, Meltzer PS. Geoquery: A bridge between the gene expression omnibus (geo) and bioconductor. *Bioinformatics*. 2007;23(14):1846-1847. doi: 10.1093/bioinformatics/btm254.

2. Z. WS, Ghamdan AE, Lee RD, et al. A single-cell and spatially resolved atlas of human breast cancers. *Nature Genetics*. 2021;53(9):1334-1347. doi: 10.1038/s41588-021-00911-1.

3. Z. WS, Ghamdan AE, Lee RD, et al. A single-cell and spatially resolved atlas of human breast cancers. *Nature Genetics*. 2021;53(9):1334-1347. doi: 10.1038/s41588-021-00911-1.

4. Vagia E, Mahalingam D, Cristofanilli M. The landscape of targeted therapies in TNBC. *Cancers (Basel)*. 2020;12(4):916. doi: 10.3390/cancers12040916.

5. Butler A, Hoffman P, Smibert P, et al. Integrating single-cell transcriptomic data across different conditions, technologies, and species. *Nature biotechnology*. 2018;36(5):411-420. doi: 10.1038/nbt.4096.

6. Korsunsky I, Millard N, Fan J, et al. Fast, sensitive and accurate integration of single-cell data with Harmony. *Nat Methods*. 2019;16(12):1289-1296. doi: 10.1038/s41592-019-0619-0.

7. Qiu X, Mao Q, Tang Y, et al. Reversed graph embedding resolves complex single-cell trajectories. *Nature methods*. 2017;14(10):979-982. doi: 10.1038/nmeth.4402.

8. Yu G, Wang LG, Han Y, et al. Clusterprofiler: An R package for comparing biological themes among gene clusters. *OMICS*. 2012;16(5):284-287. doi: 10.1089/omi.2011.0118.

9. Newman AM, Liu CL, Green MR, et al. Robust enumeration of cell subsets from tissue expression profiles. *Nature methods*. 2015;12(5):453-457. doi: 10.1038/nmeth.3337.
